# *De Novo* Transcriptome Sequencing Analysis of cDNA Library and Large-Scale Unigene Assembly in Japanese Red Pine (*Pinus densiflora*)

**DOI:** 10.3390/ijms161226139

**Published:** 2015-12-04

**Authors:** Le Liu, Shijie Zhang, Chunlan Lian

**Affiliations:** Asian Natural Environmental Science Center, The University of Tokyo, Midori-cho 1-1-8, Nishi Tokyo, Tokyo 188-0002, Japan; liu-le@anesc.u-tokyo.ac.jp (L.L.); zsjtd310@anesc.u-tokyo.ac.jp (S.Z.)

**Keywords:** *Pinus densiflora*, transcriptome sequencing, unigene assembly, simple sequence repeats, EST-SSR marker discovery

## Abstract

Japanese red pine (*Pinus densiflora*) is extensively cultivated in Japan, Korea, China, and Russia and is harvested for timber, pulpwood, garden, and paper markets. However, genetic information and molecular markers were very scarce for this species. In this study, over 51 million sequencing clean reads from *P. densiflora* mRNA were produced using Illumina paired-end sequencing technology. It yielded 83,913 unigenes with a mean length of 751 bp, of which 54,530 (64.98%) unigenes showed similarity to sequences in the NCBI database. Among which the best matches in the NCBI Nr database were *Picea sitchensis* (41.60%), *Amborella trichopoda* (9.83%), and *Pinus taeda* (4.15%). A total of 1953 putative microsatellites were identified in 1784 unigenes using MISA (MicroSAtellite) software, of which the tri-nucleotide repeats were most abundant (50.18%) and 629 EST-SSR (expressed sequence tag- simple sequence repeats) primer pairs were successfully designed. Among 20 EST-SSR primer pairs randomly chosen, 17 markers yielded amplification products of the expected size in *P. densiflora*. Our results will provide a valuable resource for gene-function analysis, germplasm identification, molecular marker-assisted breeding and resistance-related gene(s) mapping for pine for *P. densiflora*.

## 1. Introduction

*Pinus* is one of the largest genuses in the conifer family Pinaceae, with approximately 100 species [1]. As the typical specie of conifer, *Pinus densiflora* (Japanese red pine) is widely distributed and has a home range that includes Japan, Korea, China, and the extreme southeast of Russia [2]. It is widely cultivated as an ornamental tree and as construction timber for its durability, strength, and lightness [3,4]. The insect resistance of *P. densiflora* is weak, especially against the pine wilt disease which is caused by the pine wood nematode (*Bursaphelenchus xylophilus*) [5]. In China, the pine nematode disease was first discovered in Mount Zijin in 1982. Within a decade, *P. densiflora* almost completely disappeared in this area [5]. However, the control and prevention of pine wilt disease has been lagging behind and is becoming an important task for the pine ecosystem and forestry industrial system. Hence, detecting and evaluating the potential resistance genes which are used for understanding the resistance mechanism and cultivating the resistant varieties is urgently needed.

SSRs (simple sequence repeats) are excellent molecular genetic markers that could be detected in any eukaryotic genomes which both in protein-coding and non-coding regions, and have been widely applied to examine genetic diversity, germplasm identification, molecular marker-assisted breeding, and resistance-related gene(s) mapping in a broad variety of species [6,7,8]. SSRs can usually be divided into genomic SSRs (gSSR) and expressed sequence tag SSRs (EST-SSRs) [9]. In general, the development of gSSR marker applications is based on the design of species-specific primers for PCR according to the species’ genomic sequence which is costly and time-consuming. Meanwhile, for most species, the available DNA sequence data are still limited. Thus, a variety of methods have been designed to develop as many SSR primers as possible; for example, EST-SSRs are developed from cDNA sequences of different tissue sources [10]. Compared with genomic SSRs, EST-SSR markers are more attractive because they are located in the coding regions and have more cross-species transferability [10]. For example, Vendramin *et al.* [11] developed eighteen EST–SSR markers from the peach cDNA library, and all primers led to successful amplification in six other *Prunus* species. Wei *et al.* [12] successfully distinguished the polymorphism among 24 sesame accessions using forty EST-SSR primer pairs. Therefore, EST-SSRs developed from a potential specie can reduce cross-species constraints and still provide sufficient polymorphisms within related species.

During the last decade, massive sequencing platforms have become widely available, and Next Generation Sequencing (NGS), especially *de novo* transcriptome sequencing, has become more and more important in investigating the characteristics of gene transcription, regulation, and networks in model and non-model species, which has provided unprecedented opportunities for these non-model species, such as pines, lake sturgeon, and chrysanthemum [13,14,15,16], resulting in a great number of transcriptomic sequences becoming available and could be found in the National Center for Biotechnology Information (NCBI) database in the form of expressed sequence tags (ESTs). Consequently, these databases were all valuable resource for SSR marker development [15] and have greatly deepened our understanding of the resistance mechanism and disease resistance breeding of higher plants [17]. Although some ESTs have been obtained from cDNA libraries from different cells, tissues, and organs, resources from *P. densiflora* are still scarce and few SSR markers have been developed. Here, we showed that transcriptome sequencing is an attractive alternative for genetic information discovery and marker development in *P. densiflora.* Furthermore, we developed EST-SSR markers that are potentially useful for gene-function analysis, germplasm identification, molecular marker-assisted breeding, and resistance-related gene(s) mapping for *P. densiflora*.

## 2. Results and Discussion

### 2.1. Transcriptome Sequencing and De Novo Assembly

A total of 51,924,158 clean reads (44.62% GC percentage) were generated using Illumina paired-end sequencing (BioProject accession: SRS1122126, Sample: SRS1122126, Experiment: SRX1355972, Run: SRR2890864). Recently, the genome sequencing of *P. taeda* was completed successfully [18]. In the present study, *P. taeda* genome information was used to evaluate transcriptome coverage breadth by determining the total clean reads detected in our sequence collection using BLAST. We found 22.40% of the clean reads mapped to *P. taeda* (20.97% unique match and 1.43% multi-position match), with only a smaller portion (3.51%) being near perfect match (Table 1).

**Table 1:** Summary of mapping statistics to *P. taeda*.

Map to *P. taeda*	Reads Number	Percentage
Total reads	51,924,158	100.00%
Total mapped reads	11,633,342	22.40%
Perfect match	1,824,138	3.51%
Unique match	10,889,229	20.97%
Multi-position match	744,113	1.43%
Total unmapped reads	40,290,816	77.60%

**Figure 1:**
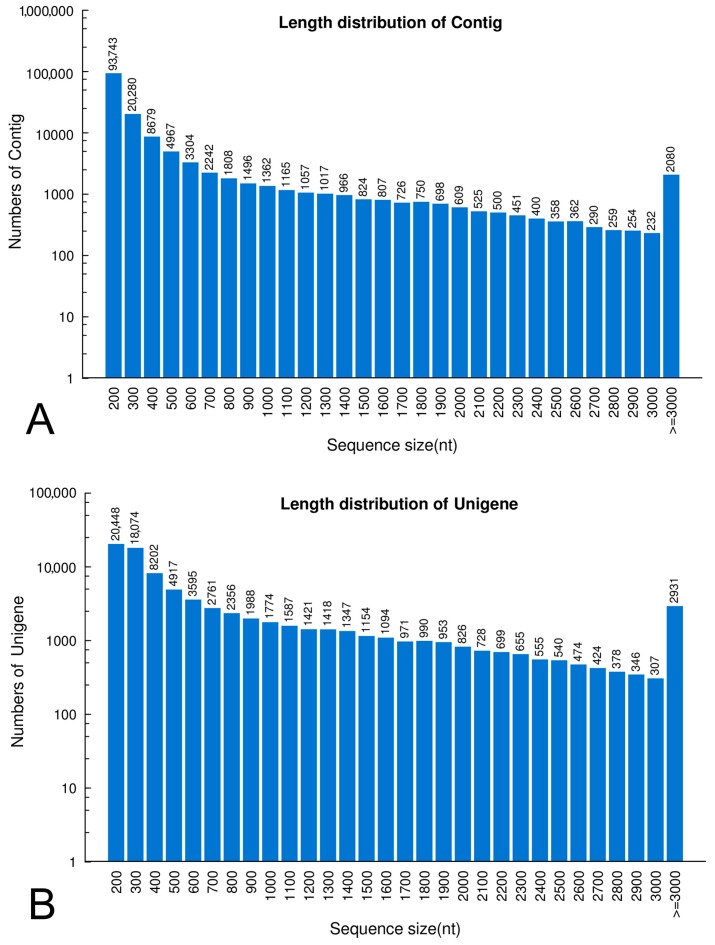
Sequence length distribution of contigs (**A**) and unigenes (**B**).

We yielded 152,211 contigs with a mean length of 392 bp (Figure 1A). Finally, a total of 83,913 unigenes were obtained after further assembly, of which 25,629 were contigs and 58,284 were singletons, with an average of 751 bp and ranged from 150 to 16,410 bp (Figure 1B).

### 2.2. Gene Annotation and Analysis

Annotation provides valuable information on the potential function of genes. A total of 45,571 (54.31%), 46,974 (55.98%), 31,040 (36.99%), 26,546 (31.64%), 23,461 (27.96%) and 15,773 (18.80%) unigenes showed high similarity to the public NCBI Nr (Non-redundant protein), Nt (Non-redundant nucleotide), Swiss-Prot (Swiss Protein), KEGG (Kyoto Encyclopedia of Genes and Genomes), GO (Gene Ontology) and COG (Clusters of Orthologous Groups) databases, respectively. However, only six unigenes had matched with previously published *P. densiflora* protein sequences in Nr protein databases (zero hits in Nt and Swiss-Prot databases), where a few ESTs from *P. densiflora* are available.

**Figure 2:**
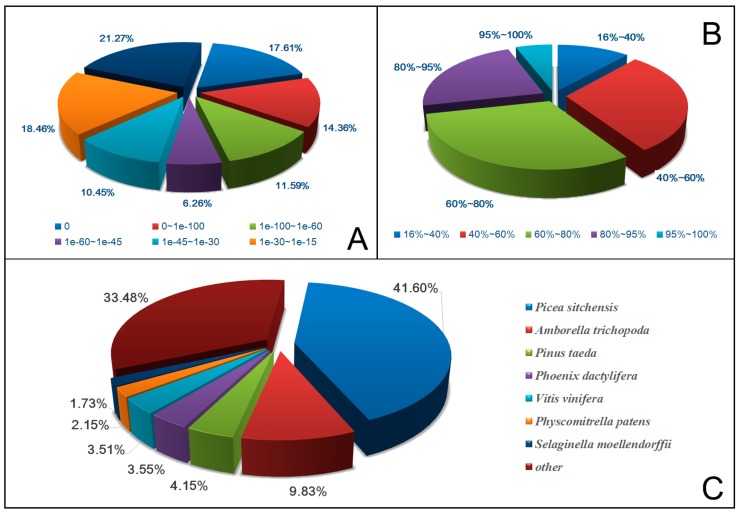
*e*-Value (**A**); similarity (**B**); and species distributions (**C**) of the unigenes.

The *e*-value distributions of the top hits in the Nr database possessed comparable patterns with 31.97% (0-1e-100) of the sequences possessing high homology (Figure 2A), while the similarity distributions of the top hits in the Nr database possessed comparable patterns with 28.49% (100%–80%) of the sequences possessing high similarity (Figure 2B). Furthermore, the best matches in the NCBI Nr database were *Picea sitchensis* (41.60%), *Amborella trichopoda* (9.83%), and *P. taeda* (4.15%) (Figure 2C). In addition, all available *P. taeda* (a related specie) cDNA databases were also used to improve the unigenes annotation. Although a total of 43,311 (51.61%) unigenes had significant hits to *P. taeda* database, there was still a considerable proportion of the unigenes were unmatched (40,602, 48.38%), therefore, further studies are required to improve the annotations.

**Figure 3:**
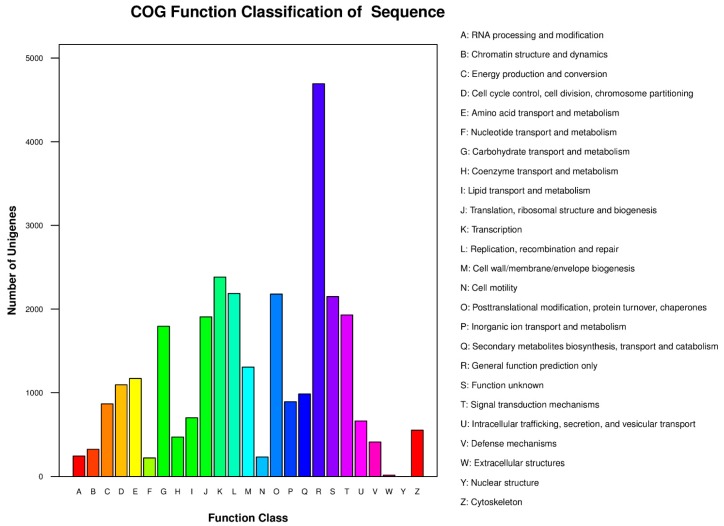
COG function classification of the unigenes.

All unigenes were also compared to the proteins in the COG database, and a total of 29,358 sequences were assigned to pre-existing COGs. Among the 25 COG categories assigned, the largest group was the cluster for general function prediction only (4693, 15.99%), followed by transcription (2382, 8.11%), replication, recombination, and repair (2186, 7.45%), posttranslational modification, protein turnover, and chaperones (2179, 7.42%), function unknown (2149, 7.32%), signal transduction mechanisms (1929, 6.57%) and translation, ribosomal structure, and biogenesis (1906, 6.49%) (Figure 3).

**Figure 4:**
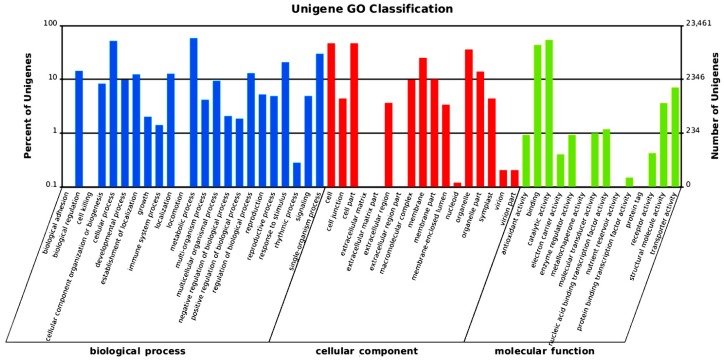
GO functional distribution of sequences. Biological process (blue); cellular component (red); and molecular function (green).

After Nr annotation, a total of 62,967 annotated unigenes were sorted into the category of biological processes. Within the biological process ontology, the two most common types of genes were metabolic processes (13,704, 21.76%) and cellular processes (12,215, 19.40%). Among the cellular component unigenes, the three most common types were localized to the cell (10,988, 22.86%), cell part (10,988, 22.86%), and organelle (8434, 17.54%) (Figure 4).

Although the assigned molecular functions of the unigenes covered a wide range of GO categories, catalytic activity (12,768, 47.75%) and binding (10,254, 38.34%) proteins made up the majority, while receptor activity, antioxidant activity, enzyme regulator activity, molecular transducer activity, nucleic acid binding transcription factor activity, structural molecule activity, and transporter activity proteins together made up only 13.33% (Figure 4). Notably, compared with other species, such as *Dendrocalamus*, *Physcomitrella*, and *Chyrsanthemum* [15,19,20], the COG function classification was largely identical, as COG protein was inherited from the last common ancestor, however, in the different GO classes, the number of genes showed a wide variation which may reflect the metabolic or physiological bias under the different environment.

Due to a great improvement of efficiency, cost-effectiveness and accuracy, next-generation sequencing (NGS) has been widely applied to obtain large-scale unique EST sequences, especially in non-model organisms such as *Dendrocalamus latiflorus*, *Physcomitrella*, and *Chyrsanthemum* [15,20,21], providing valuable sequences for gene discovery and annotation and comparative genomics [22]. Our EST resources for *P. densiflora* consisted of 83,913 unique sequences and were comparable to 3136 ESTs available in the NCBI database. Notably, many genes failed to match any functional annotation, which is possibly due to the shorter sequence length. It is also noteworthy that many unigenes matched genes with unique annotations. These could be used to produce single nucleotide polymorphism markers and identify alternative gene splicing, homologous genes, and gene families [23,24,25,26]. With a cut-off *e*-value <10^−5^, 56% of unigenes could match to known proteins. Approximately 75.04% of the annotated unigenes could be found in the GO category “biological process”, suggesting a comprehensive diversity of unigenes we obtained, and these resources will be available for identifying resistance-related gene(s) and large-scale gene expression analysis in future studies.

In the transcriptome sequencing, Guo *et al.* [27] reported that assembly and annotation of watermelon, nearly two-thirds of sequences matched proteins of the genome-sequenced cucumber, which is the most closely related species to watermelon. In the current study, a considerable proportion of the potential unigenes showed high sequence similarity to *Pi. sitchensis* (41.60%) and *A. trichopoda* (9.83%) rather than *P. taeda* (4.15%, in spite of the same genus to *P. densiflora*) and only six sequences hit with previously published *P. densiflora* nucleotide sequences. This is because, in the database, only a few ESTs of *P. densiflora* or its closely related species were released, whereas larger amounts of cDNA or protein sequences information of *Pi. sitchensis* and *A. trichopoda* were released. Meanwhile, there was still a considerable proportion of the unigenes that were unmatched (48.38%) when *P. taeda* was used as the cDNA reference database; therefore, further studies were required to improve the unigene annotations.

### 2.3. SSR Motifs Characterization and SSR Markers Development

From the 83,913 present unigenes, a total of 1953 putative microsatellites (mono-nucleotide repeats not included) were detected in 1784 unigenes (167 with more than one SSR). The frequency of EST–SSRs which we observed in the present unigenes was only 2.33%. Among the different EST-SSR repeat types determined, the most abundant were tri-nucleotide repeats (980, 50.18%), followed by dinucleotide repeats (703, 36.00%), hexa-nucleotide repeats (163, 8.35%), penta-nucleotide repeats (77, 3.94%), and quad-nucleotide repeats (30, 1.54%) (Table 2). The maximum of repeats was 12 for di-nucleotides, while 18, six, five, and 13 for tri-nucleotides, tetra-nucleotides, penta-nucleotides, and hexa-nucleotides, respectively. Among these, five tandem repeats were the most common repeat number (683, 34.97%) in tri-nucleotides, followed by six tandem repeats (298, 15.26%) in di-nucleotides, six tandem repeats (192, 9.83%) in tri-nucleotides, four and seven (142, 7.27%) tandem repeats in hexa-nucleotides and di-nucleotides (Table 2).

**Table 2:** Distribution of SSR Size.

Number of Repeats	Di-Nucleotide Repeats	Tri-Nucleotide Repeats	Quad-Nucleotide Repeat	Penta-Nucleotide Repeats	Hexa-Nucleotide Repeats
**4**	-	-	-	65	142
**5**	-	683	29	12	10
**6**	298	192	1	0	4
**7**	142	86	0	0	3
**8**	105	16	0	0	0
**9**	56	1	0	0	2
**10**	58	0	0	0	0
**11**	41	1	0	0	1
**12**	3	0	0	0	0
**13**	0	0	0	0	1
**14**	0	0	0	0	0
**15**	0	0	0	0	0
**16**	0	0	0	0	0
**17**	0	0	0	0	0
**18**	0	1	0	0	0
**19**	0	0	0	0	0
**20**	0	0	0	0	0
**22**	0	0	0	0	0
**23**	0	0	0	0	0
**SubTotal**	703	980	30	77	163

**Figure 5:**
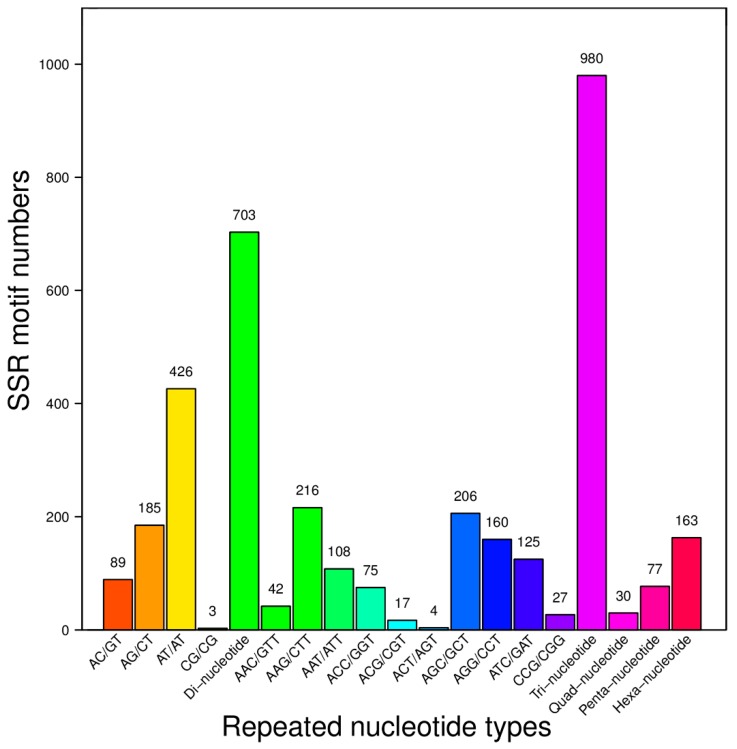
Frequencies of different repeat motifs in the EST-SSRs.

Among the various SSRs, more than 140 motif sequence types were identified (considering sequence complementary) (Table S1), and the AT/AT motif was the most frequent (426, 21.81%) followed by AAG/CTT (216, 11.06%), AGC/CTG (181, 9.27%), AG/CT (162, 8.29%), AGG/CCT (131, 6.71%), and ATC/GAT (125, 6.40%) (Figure 5).

**Figure 6:**
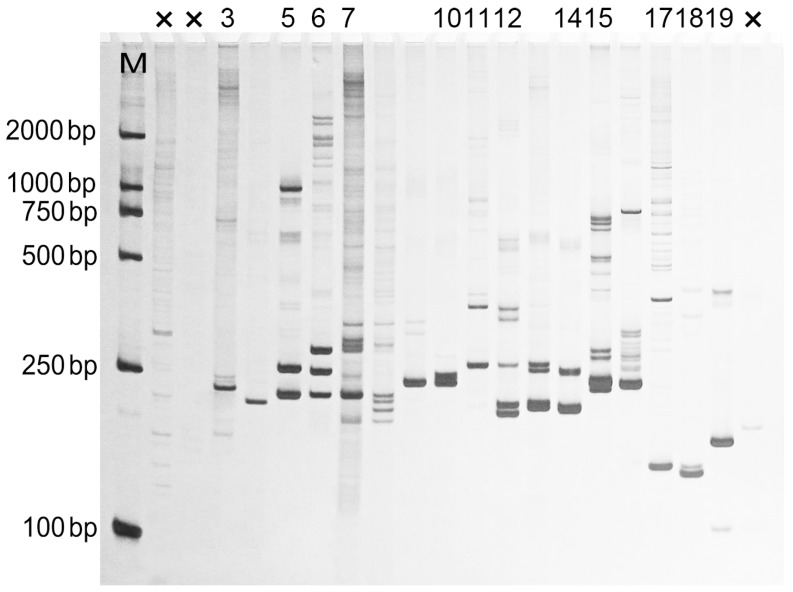
PCR amplification using 20 primer pairs randomly selected in *P. densiflora*. ”×”: void amplification. M: DNA Marker DL2000.

Finally, of the 1953 putative SSR motifs identified, 629 pairs PCR primers were designed successfully (Table S2). The flanking sequences of the left SSR loci were different to be used for primer design. To test whether the potential EST–SSR loci were available in population genetics, 20 out of 629 primer pairs were randomly selected and synthesized (Table S3). Of these primer pairs, 17 successfully amplified genomic DNA of *P. densiflora*, and all of those yielded amplification products of the expected size (100–300 bp) (Figure 6), implying that these primers could be used for molecular-assisted breeding and other marker-related studies in *P. densiflora* in the future.

**Figure 7:**
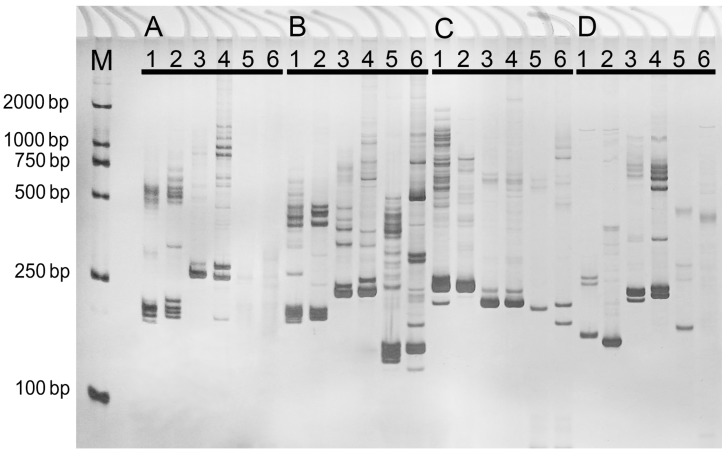
PCR amplification using four primer pairs (**A**–**D**) in six related species. 1: *P. densiflora*, 2: *P. taeda*, 3: *Pi. sitchensis*, 4: *Pi. asperata*, 5: *Ab. Firma*, and 6: *Ab. Fabri*. M: DNA Marker DL2000.

As derived from coding regions, EST-SSR markers were more likely to be transferable across species compared with gSSR markers [15]. In our study, four primer pairs which showed a high similarity to *Pi. sitchensis* or *P. taeda* sequences were selected (Table S4) to investigate polymorphisms of *P. densiflora*, *P. taeda*, *Pi. sitchensis*, *Pi. asperata*, *Abies firma*, and *Abies fabri*. The results showed that markers developed in the present study had high transferability. For example, we did not find any bands using primer pairs A in *Ab. firma* and *Ab. fabri* (Figure 7A), and using primer pairs D in *Ab. fabri*, while nearly all species showed a high genetic polymorphism using primer pairs B and C within three genuses.

As the most promising co-dominant markers, identification of these repeating genomic modifications is still a tedious and iterative process [10,28]. For example, we previously reported only six SSR markers using a microsatellite-enriched library that can be used to estimate effective gene transfer in conifers by comparing the segregation in the parental and offspring groups [29]. Transcriptome analysis could provide a high throughput method for the development of SSR markers for *P. densiflora*. In our current study, we identified 1953 putative EST-SSRs and developed 629 EST-SSR primers based on the present transcriptome. To our knowledge, our study is the first attempt on the development of numerous EST-SSRs for *P. densiflora*, and it is also a fast and cost-effective approach to microsatellite discovery and molecular marker development for plants, especially for those with large genomes.

EST-SSR markers are ideal markers whose polymorphisms might be caused by genetic variation in coding regions and are concerned with mutation and phenotypic variation [30,31]. EST-SSR markers also have a higher cross-species transferability than genomic SSR markers across diverse germplasms [32]. In the present study, the most abundant nucleotide repeat was the tri-nucleotide repeat (35%), and di-nucleotide (32%) repeats ranked second. Similar relative frequency of these nucleotide repeats on transcriptome level has been also detected in many other species including *Vigna radiata* [33], *Medicago trunculata* [34], and *Chrysanthemum* [15]*.* Note that some unrecognized intron splice sites or large introns in the genome might disrupt amplification of primer pairs. Nonetheless, in the present study, we still obtained a large number of polymorphism band among different species using the selected primer pairs (Figure 7). Therefore, markers we developed in this study will be of benefit for gene-function analysis, germplasm identification, molecular marker-assisted breeding, and resistance-related gene(s) mapping for *Pinus* in the near future.

## 3. Experimental Section

### 3.1. Plant Material and RNA Extraction

Plant materials are maintained in the University of Tokyo Tanashi Forest, located in suburban Tokyo, Japan. *P. densiflora* at vegetative stages, *i.e.*, 90 days old from the seeds of trees with sowing and growing in the greenhouse, were harvested from a pool of three plants (the whole seeding, more than one sample pool were harvested) and used in the large-scale transcript sequence analysis.

Extracting RNA from the whole seedling using the Total RNA Isolation System (Takara, Japan). RNA quality (RIN > 8.5 and 28S:18S > 1.5) was confirmed by RNA Nanochip (Agilent, Santa Clara, CA, USA). The RNA was quantified by spectrophotometer (NanoDrop ND-1000, Wilmington, DE, USA) with the standard 1.8 ≤ OD260/280 ≤ 2.2, OD260/230 ≥ 1.8. More than 20 μg of RNA (samples contributed equally) was used for the single cDNA library preparation.

### 3.2. Illumina Sequencing

Illumina sequencing was implemented at the BGI Company, Shenzhen Genomics Institute (Shenzhen, China). The library we prepared using an Illumina HiSeq™ 2000 (Illumina, Dedham, MA, USA).

### 3.3. Data Output and De Novo Assembly

Data output from the sequencing platform was named as raw reads and stored in FASTQ format, among which, adapters, >5% unknown nucleotides, and low quality bases in raw reads were removed and a stringent filtering process was performed.

We preformed *de novo* transcriptome assembly using Trinity software (Campton, NH, USA) [35]. Sequence direction was judged by BLASTX alignment using non-redundant protein (Nr), non-redundant nucleotide (Nt), Swiss-Prot, Gene Ontology (GO), and Cluster of Orthologous Groups (COG) database (in priority order). The rest sequence direction was decided by ESTScan [36].

### 3.4. Structural and Functional Annotation

To provide further information on the potential functions of the unigenes, annotation information was assigned from the protein, COG, and Gene Ontology (GO) databases. The BLASTX algorithm [37] with a cut-off *e*-value <10^−5^ was employed to search for similar sequences in several public databases (*i.e.*, Nr and Swiss-Prot protein databases) to retrieve the most closely-related sequence and its functional annotations. Based on the Nr annotations, GO annotations of the unigenes were obtained using the Blast2GO program [38]. After getting GO annotations for every unigene, we further assigned GO functional classifications for these unigenes and distributed gene functions at the macro level using WEGO software [39].

### 3.5. SSR Loci Identification and Primer Pairs Design

SSR loci of unigenes were identified by MISA, which was used to set the standard to identify SSR markers. To amplify a sequence with appropriate length (>100 bp) on the basis of ensuring the quality of the primer, only sequences with the length of both ends of SSRs greater than 150 bp were used to design these primers. In this study, Primer 3-2.3.4 software was employed to design PCR primers for the conserved flanking regions of the SSRs (at least six repeats for di-, five repeats for tri-, four repeats for tetra-, and three repeats for penta- and hexa-nucleotide motifs). A pair of primer was designed based on (1) a primer length of 18–28 bp, with 23 bp as the optimum; (2) a melting temperature between 55 and 65 °C, with a maximum discrepancy of 4 °C among primers; (3) no SSRs in the primer; (4) a control PCR product size of 100 to 300 bp; and (5) removal of the primers that aligned to more than one unigene (Table S2) [12,15].

### 3.6. EST-SSR Screening

Genomic DNA of *P. densiflora* was extracted from sampled leaves using the cetyltrimethyl ammonium bromide (CTAB) method [40]. DNA was diluted to 10 ng/μL and stored at −20 °C before use. Twenty primer pairs were randomly chosen to check their effectiveness (Table S3). Four primer pairs, which showed a high similarity to *Pi. sitchensis* or *P. taeda* sequences, were selected to investigate the polymorphisms of *P. densiflora*, *P. taeda*, *Pi. sitchensis*, *Pi. asperata*, *Ab. Firma*, and *Ab. fabri*. PCR conditions procedure was performed according to Wang *et al.* [15]. PCR products were loaded on 6% polyacrylamide gels (acrylamide/bisacrylamide ratio, 19:1, containing 7.5 M urea, buffered with 1× Tris–borate–EDTA, pH = 7.8) and separated. Bands were recorded as “1” (clearly visible) or “0” (absent) with each primer pair [41].

## 4. Conclusions

A total of 83,913 unigenes were assembled and 1953 EST-SSRs were identified in 1784 unigenes. Among these, 629 primer pairs were successfully designed using professional software and characterized as potential molecular markers. These unigenes and EST-SSR markers developed in this study could provide a valuable resource for gene-function analysis, germplasm identification, molecular marker-assisted breeding, and resistance-related gene(s) mapping for pine. For all we know, our study is the first attempt for the construction of a full-length cDNA library and NGS sequencing and large collection of EST-SSR markers for *P. densiflora.*

## Supplementary Materials

Supplementary materials can be found at http://www.mdpi.com/1422-0067/16/12/26139/s1.
